# Explainable Deep Learning-Based Feature Selection and Intrusion Detection Method on the Internet of Things

**DOI:** 10.3390/s24165223

**Published:** 2024-08-12

**Authors:** Xuejiao Chen, Minyao Liu, Zixuan Wang, Yun Wang

**Affiliations:** 1 School of Communications, Nanjing Vocational College of Information Technology, Nanjing 210023, China; 2 School of Modern Posts, Nanjing University of Posts & Telecommunications, Nanjing 210003, China; 1222097606@njupt.edu.cn (M.L.); 2020070135@njupt.edu.cn (Z.W.); johnchen0825@outlook.com (Y.W.)

**Keywords:** model interpretability, feature selection, deep learning, random forest, convolutional neural network, information gain, RFE, SHAP

## Abstract

With the rapid advancement of the Internet of Things, network security has garnered increasing attention from researchers. Applying deep learning (DL) has significantly enhanced the performance of Network Intrusion Detection Systems (NIDSs). However, due to its complexity and “black box” problem, deploying DL-based NIDS models in practical scenarios poses several challenges, including model interpretability and being lightweight. Feature selection (FS) in DL models plays a crucial role in minimizing model parameters and decreasing computational overheads while enhancing NIDS performance. Hence, selecting effective features remains a pivotal concern for NIDSs. In light of this, this paper proposes an interpretable feature selection method for encrypted traffic intrusion detection based on SHAP and causality principles. This approach utilizes the results of model interpretation for feature selection to reduce feature count while ensuring model reliability. We evaluate and validate our proposed method on two public network traffic datasets, CICIDS2017 and NSL-KDD, employing both a CNN and a random forest (RF). Experimental results demonstrate superior performance achieved by our proposed method.

## 1. Introduction

As Internet of Things (IoT) technology progresses, an increasing array of IoT devices offering various innovative services and applications is being developed. This array includes sensors, actuators, multiple appliances, and consumer-focused devices such as smartphones, intelligent home devices, and domestic IoT sensors. These devices are often powered by lightweight operating systems, which may result in constrained processing power and, as a consequence, diminished security measures. Moreover, their susceptibility to network attacks is heightened by limited communication functions and volatile connections. The spectrum of cyberthreats they face encompasses but is not confined to botnets, forceful entry attempts, Denial-of-Service (DoS), and Distributed Denial-of-Service (DDoS) assaults, along with web-based attacks. Such incursions could compromise IoT devices’ functionality, expose sensitive data, or even allow malevolent entities to seize control remotely. Consequently, deploying Network Intrusion Detection Systems (NIDSs) specifically designed for IoT environments is crucial for their protection.

In recent decades, a variety of machine learning (ML) techniques have been employed in the realm of NIDSs, including but not limited to decision trees (DTs) [1], Support Vector Machines (SVMs) [2], and random forests (RFs) [3]. Deep learning (DL), a subset of ML, has been increasingly integrated into network security [4]. With its superior capability to discern complex, high-dimensional attributes and to accommodate extensive network datasets, DL has been instrumental in mitigating the traditionally lower precision associated with ML. Numerous studies have documented enhanced outcomes by applying both ML and DL methodologies.

The mainstream DL neural network structures are often complex, with many layers and a large number of nodes. For example, Alexie [5], a convolutional neural network designed for classification tasks, made a breakthrough in the ILSVRC competition in the early stage. It was composed of five convolution layers and three full connection layers, with more than 60 million parameters and a model size of more than 240 MB. In addition, the widely used model VGG16 [6] in the industry has 144 million parameters, and the model size exceeds 500 MB. Resnet-152 [7] has 57 million parameters, and the model size reaches 230 MB. Using the above model to classify network packets containing video and audio requires more than 10 billion floating-point calculations. In addition, because Resnet-series models have complex branch structures, although their numbers of parameters are smaller than those of VGG models with flat structures, they take longer in practical training and inferring. It can be seen that the storage and computing costs of mainstream DL models are too high for real-time applications with strict delay constraints.

With the popularity of IoT devices and the development of edge computing, in an actual scenario, many NIDS models are deployed in edge devices, such as edge gateways and switches. In traffic detection tasks involving gigabit bandwidths, edge devices constrained by resource limitations will likely experience significant packet loss and delays. Such devices fail to satisfy the real-time demands of intrusion detection systems. Consequently, model compression and the minimization of detection latency are critical to address these challenges.

Feature selection (FS) is a technique for reducing data dimensions, primarily focusing on isolating the most advantageous attributes from the initial dataset. This process diminishes the data’s dimensionality and the complexity of learning tasks, thereby enhancing the model’s efficiency. Despite the efficacy of conventional statistical-based feature selection methods, their application process remains intricate. The computational load escalates exponentially in terms of the volume of data and the breadth of feature dimensions, presenting practical usage challenges.

Drawing on the concept of causality within ML, this study introduces an explainable artificial intelligence and feature selection method for NIDSs on the IoT. The paper’s contributions are outlined as follows:

1. The paper introduces a feature selection approach for an intrusion detection model that handles encrypted traffic, emphasizing the model’s interpretability. Initially, an explanation of the model, which incorporates the original features, is provided to determine each feature’s degree of contribution. Subsequently, a subset of features with a high degree of contribution is chosen as the most favorable subset, in alignment with specific requirements. Ultimately, this optimal set of features is employed to refine and retrain the enhanced intrusion detection model.
2. The study conducts an experimental assessment of the suggested FS technique using two standard classifiers, a convolutional neural network (CNN) and a random forest (RF), on two widely recognized datasets: NSL-KDD and CICIDS2017. When juxtaposed with two state-of-the-art (SOTA) feature selection strategies, namely information gain (IG) [8] and Recursive Feature Elimination (RFE) [9], the findings indicate the superior efficacy of the proposed FS method.

The subsequent sections of this paper are structured in the following manner: Section 2 delineates the work related to this study. Section 3 details the feature selection approach proposed for encrypted traffic intrusion detection, emphasizing the interpretability of the model. Section 4 outlines the experimental framework and the outcomes of the feature extraction process. Section 5 analyzes the findings from the comparative experiments. Finally, Section 6 summarizes the paper and offers insights into future directions.

## 2. Related Work

### 2.1. NIDSs Based on DL and ML

Conventional encrypted traffic intrusion detection techniques have developed through three distinct phases: ports, payloads, and traffic statistics. Yet, the advent of port masking, random ports, and tunneling technologies have rapidly rendered port-centric detection methods obsolete. Furthermore, DPI (Deep Packet Inspection) approaches, reliant on payload characteristics, fall short in addressing encrypted traffic detection due to their dependency on content matching within packets. Therefore, ML methods for model training using flow statistical features or timing features emerged, including Naive Bayes (NB) [10], SVMs [11], DTs [12], RFs [13], KNN, K-means.

Addressing the limitations of conventional ML techniques in the automatic extraction and selection of traffic features, the research community has pivoted to deep learning approaches such as multi-layer perceptrons (MLP) [14], CNNs [15], and Recurrent Neural Networks (RNNs) [16]. These networks undergo supervised training to autonomously discern the temporal and spatial attributes of traffic, thereby enhancing the precision of encrypted traffic detection. Zhou [17] introduced an NIDS strategy utilizing a Generative Adversarial Network for the IIoT, which demonstrated commendable accuracy. Within intelligent city frameworks, Elsaeidy [18] employed restricted Boltzmann machines, which were adept at extracting intricate features from network traffic and leveraging these features to detect a range of DDoS attacks. DL methods excel in autonomously extracting features and recognizing complex, high-level patterns, thus improving detection capabilities.

However, whether ML or DL, most researchers still use the original features provided by the dataset for training when designing intrusion detection models. Even though some features are selected, they are still based on manual or statistical methods. When facing extensive traffic data, the selected features may change, and the detection time significantly increases.

### 2.2. Feature Selection and Model Interpretability

At present, feature selection algorithms can be categorized into three main types: global optimal, random, and sequence search. The process of global optimal search entails identifying the most advantageous subset of features from the initial collection, typically employing methods such as exhaustive search or branch-and-bound techniques [19]. These approaches work well for low-dimensional feature sets but become computationally expensive as the feature dimensions increase. The random search algorithm randomly selects feature subsets and applies different processing methods. One approach injects randomness into the sequence search, as seen in the simulated annealing algorithm [20]. The other method is entirely random, often called the “completely random” approach. While random search introduces uncertainty and yields diverse feature subsets, it helps prevent local optimization and approximates the optimal solution. Frequently employed optimization methods encompass particle swarm optimization [21], ant colony optimization [22], and genetic algorithms [23]. In contrast to global search, sequence search does not ensure the discovery of the best subset of features. It evolved from greedy algorithms and includes sequential forward selection, sequential backward selection, and bidirectional search strategies. Although sequence search is efficient, the resulting feature subset may be locally optimal. Some feature selection methods integrate model recognition results during selection, iterating through features to obtain an optimal subset and model. However, this approach tends to be less efficient.

Deep learning fundamentally differs from machine learning due to its inherent self-explanatory ability. However, most deep learning models exhibit high complexity, numerous parameters, and limited transparency—they are often called “black boxes”. Understanding the decision-making mechanism of these “end-to-end” models poses challenges, hindering their practical deployment. Researchers have proposed various methods [24,25] to enhance the interpretability of deep learning models. These approaches fall into three categories: model visualization, model transformation interpretation, and feature significance analysis. Model visualization directly visualizes weight parameters, neural network neurons, or feature detectors [26,27,28]. While weight visualization provides insights into the model’s final prediction contribution, it lacks universality. Neurons and feature detectors can also be visualized to reveal input feature changes, but achieving general interpretability remains challenging. Model interpretability involves examining parameters or feature statistics within the black-box model [29,30]. For instance, using interpretable decision models or sparse linear approximations helps establish relationships between inputs and outputs, achieving interpretable migration. However, this approach does not consider the black-box model’s internal parameters and relies on direct “end-to-end” approximations. The feature significance analysis method summarizes and counts each feature according to the decision results and returns a quantitative index, such as the importance of the feature [31,32,33]. In addition, the statistical information of feature significance can be visualized, such as the feature significance map that intuitively shows the essential features. The feature statistical analysis method mainly interprets the DL model from the feature level, and the feature is used as a bridge between the interpretability and the model. In these papers, the feature significance analysis method is applied to the interpretability of the encrypted traffic identification model.

## 3. Methodology

### 3.1. The Framework of Our Explainable Artificial Intelligence and Feature Selection Method

**Figure 1:**
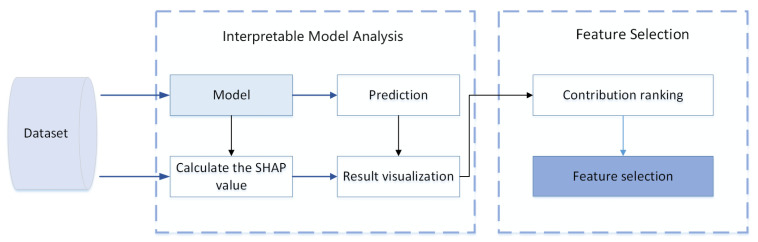
The structure of our explainable artificial intelligence and feature selection method.

The structure of the method proposed in this study is depicted in Figure 1 and primarily consists of two stages: (1) analysis of the model interpretability and (2) the selection of features.

1. Interpretable model analysis: Initially, the NIDS model undergoes training using the datasets to enhance its predictive accuracy. Following this, the Shapley value [34] for each feature within the model is computed, leading to the generation of a visual representations of the results.
2. Feature selection: according to domain expert experience, the best feature subset of compliance with causality is selected from the pre-ranking features, and the feature selection task is completed.

### 3.2. Model Interpretability

#### 3.2.1. SHAP Value

We introduce a framework for a DL-based NIDS that leverages SHAP values. These values are instrumental in measuring each feature’s impact on model predictions. The core concept involves computing the incremental SHAP value as features are incorporated into the model, thereby facilitating an understanding of feature significance through SHAP values. Originating from game theory, the SHAP value assesses individual contributions to collective success in cooperative scenarios. This method is suitable for clarifying predictions within machine learning frameworks, as the Shapley value quantifies the average impact of features on the predictions over all conceivable combinations:

(1) $$\phi_{i}\left(f,x^{\prime }\right)=\sum_{z^{\prime }\subseteq \left\{x_{1}^{\prime },\ldots ,x_{n}^{\prime }\right\}\setminus \left\{x_{i}^{\prime }\right\}}\frac{\left|z^{\prime }\right|!\left(M-\left|z^{\prime }\right|-1\right)!}{M!}*\left[f\left(z^{\prime }\cup x_{i}^{\prime }\right)-f\left(z^{\prime }\right)\right]$$

- $x^{\prime }=\left\{x_{1}^{\prime },\ldots ,x_{n}^{\prime }\right\}$ is the eigenvalue vector of the instance to be interpreted; $x_{i}^{\prime }$ is the *i*th eigenvalue in vector $x^{\prime }$.
- $z^{\prime }$ is a subset of the features used in the model.
- *M* is the number of eigenvalues.
- $f\left(z^{\prime }\right)$ is the predicted value of $z^{\prime }$. When $f\left(z^{\prime }\right)$ is calculated, the *i*th feature is masked, and then the random instance or the random value of the *i*th feature is drawn from the dataset to simulate the *i*th feature.
- $\phi_{i}\left(f,x^{\prime }\right)$ calculates the weighted average of the marginal contributions of variable $x_{i}^{\prime }$ across all possible subsets $z^{\prime }$. This method ensures that each variable’s contribution is fairly evaluated, independent of its order of appearance with respect to other variables.

The SHAP value computation is equitable for every feature, allowing for a global assessment by comparing a feature’s SHAP value across various samples. SHAP, an additive explanatory model, draws from the Shapley value concept. It assigns a SHAP value to each feature within a sample, corresponding to the model’s prediction for that sample. If we represent the *i*th sample by $X_{i}$, the *j*th feature of this sample by $\chi_{ij}$, the predicted value by the model for this sample as $Y_{i}$, and the baseline value of the model (usually the mean of the target variables across all samples) as $Y_{\mathrm{base}\;}$, the SHAP value adheres to the ensuing equation:

(2) $$Y_{i}=Y_{\mathrm{base}\;}+f\left(\chi_{i1}\right)+f\left(\chi_{i2}\right)+f\left(\chi_{i3}\right)+f\left(\chi_{i4}\right)+\cdots +f\left(\chi_{ik}\right)$$

The SHAP value, denoted as $f\left(\chi_{ik}\right)$, represents the contribution of the *k*th feature in the *i*th sample, $\chi_{ik}$, to the model’s final prediction, $Y_{i}$. A positive SHAP value, $f\left(\chi_{ik}\right)>$ 0, signifies that the feature has a beneficial impact on the predicted outcome, enhancing it. Conversely, a negative SHAP value indicates a detrimental effect, diminishing the predicted outcome. The primary advantage of SHAP values is their ability to capture feature impact within each sample, revealing positive and negative correlations. By statistically summarizing the contribution of each characteristic and ranking them, we obtain a visual representation. Ultimately, leveraging expert insights, we select the optimal feature subset that adheres to causality from the pre-ranked features, thereby facilitating the feature selection task.

#### 3.2.2. The Architecture of Model Interpretability

**Figure 2:**
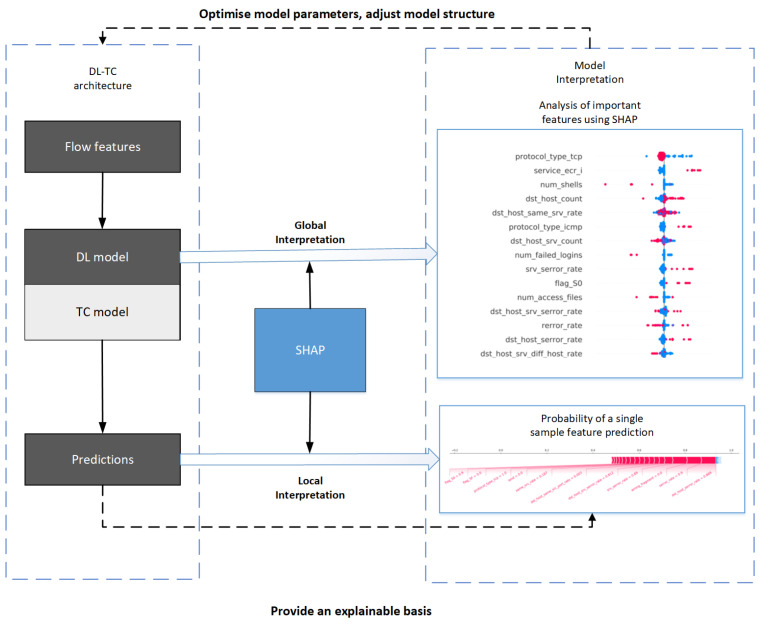
The proposed method structure.

As shown in Figure 2, the architecture for model interpretation includes two principal elements. The traditional configuration of the traffic classification model is presented on the left side. On the right side, the depicted procedure enhances model interpretability and optimization through modifications to the model’s architecture and settings. This approach integrates interpretations on both the local and global scales. In Figure 2, the two block diagrams in the right half part illustrate the interpretable model analysis in the visualized way called force plot, in which the right top corner presents the result of global interpretation, accordingly, the right bottom one is local interpretation. In the two force plots, the red indicates the feature importance as POSITIVE; otherwise, the blue one is NEGATIVE. The significance of these features can be distinctly shown in such a visualized manner.

Local interpretation involves computing and visually representing the contribution of each feature to the predicted outcome for individual data instances. The formula for this calculation is expressed as:

(3) $$y_{i}=y_{\mathrm{base}\;}+f\left(x_{i1}\right)+f\left(x_{i2}\right)+f\left(x_{i3}\right)+f\left(x_{i4}\right)+\cdots +f\left(x_{ik}\right)$$

In this expression, $y_{i}$ signifies the value predicted by the model for sample $x_{i}$, while $y_{base}$ represents the average of the evaluated values for all samples.

For the global interpretation, the initial step involves computing a matrix where SHAP values for features are arrayed, assigning each instance to a row and each feature to a column. In conventional global interpretation practices, the contribution of feature *j* is ascertained by aggregating the average Shapley value of feature *j* across all instances, as delineated in Equation (4). Subsequently, the SHAP values are arranged in descending sequence to determine the significance of the features within the model.

(4) $$f\left(x_{j}\right)=\sum_{i=1}^{M}f\left(x_{ij}\right)$$

### 3.3. Validation Method

**Figure 3:**
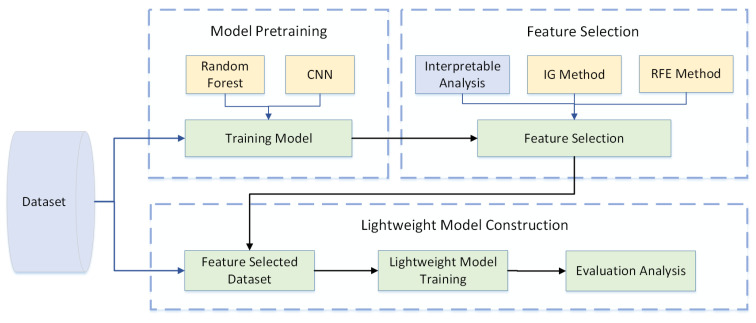
The validation method.

To detect the effect of feature selection, a validation method was designed. The methodology outlined in Figure 3 is comprised of a tripartite process: model pretraining, feature selection, and lightweight model construction.

#### 3.3.1. Model Pretraining

**Figure 4:**
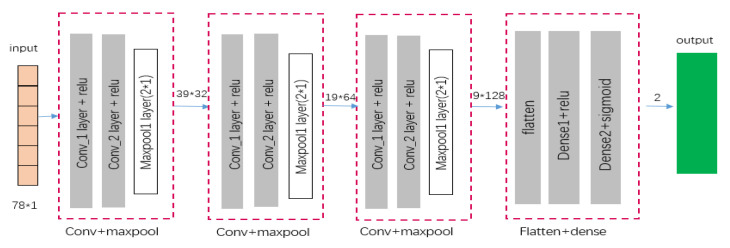
The CNN model structure.

This study utilized a CNN and an RF for both experimental and comparative analyses. The random forest algorithm, an ensemble of multiple decision trees, processes each tree as a classifier to produce N possible classifications for a given input. The random forest then aggregates these outcomes, with the most frequently occurring classification being selected as the final decision. A multi-layered, supervised learning neural network characterizes CNN’s architecture. Within its hidden layers, convolutional and pooling layers act as fundamental components that facilitate the extraction of features. To reduce the loss function, the network model applies gradient descent, fine-tuning the weights within the layers to improve prediction accuracy. CNN’s lower hidden layers are composed of convolutional and max-pooling layers. In contrast, the upper layers include the fully connected layer and the logistic regression classifier, akin to those found in a standard multi-layer perceptron. The CNN model structure employed in this research is illustrated in Figure 4.

#### 3.3.2. Feature Selection

To validate the feasibility of the model-based interpretable feature selection method, we chose the Univariate FS (UFS), Recursive Feature Elimination (RFE), Random Forest Importance (RFI), Forward Sequential FS (FSFS), and information gain (IG) for comparison.

1. Univariate FS

Univariate filters evaluate and rank a single feature according to a certain criterion, while multivariate filters evaluate the entire feature space. Univariate FS builds one decision tree per feature to predict the target, then makes predictions and ranks the features according to the machine learning metric (roc-auc or mse).

2. Recursive Feature Elimination

In a similar vein, RFE utilizes a recursive approach to progressively diminish the feature set’s dimensions, thereby isolating the essential features.

(1) RFE assigns a weight to each feature, followed by the employment of a predictive model for training purposes utilizing these initial features.

(2) After the acquisition of the feature’s weight, the absolute values of these weights are computed, and the one with the smallest absolute value is discarded.

(3) This recursive process is perpetuated until the number of features that remain corresponds to the predetermined requisite.

3. Random Forest Importance

Selecting features by using tree derived feature importance is a very straightforward, fast, and generally accurate way of selecting good features for machine learning. For classification, the measure of impurity is either the Gini impurity or the information gain/entropy. For regression, the measure of impurity is variance. Therefore, when training a tree, it is possible to compute how much each feature decreases the impurity. The more a feature decreases the impurity, the more important the feature is. In random forests, the impurity decrease from each feature can be averaged across trees to determine the final importance of the variable.

4. Forward Sequential FS

Wrappers use a search strategy to search through the space of possible feature subsets and evaluate each subset by the quality of the performance on an ML algorithm. Step-forward feature selection starts by evaluating all features individually and selects the one that generates the best performing algorithm, according to a pre-set evaluation criterion. In the second step, it evaluates all possible combinations of the selected feature and a second feature and selects the pair that produce the best performing algorithm based on the same pre-set criterion.

5. Information gain

Entropy quantifies the unpredictability of random variables and forms the foundation of IG. The entropy for random variables is computed in the following manner: Consider X to be a discrete random variable that assumes a limited set of possible values, with the probability distribution given by:

(5) $$\begin{array}{c} P\left(X=x_{i}\right)=p_{i},i=1,2,3,\ldots n \end{array}$$

Then, the entropy of the random variable X is defined as:

(6) $$\begin{array}{c} H\left(X\right)=-\sum_{1}^{n}p_{i}*\log p_{i} \end{array}$$

The calculation of the conditional entropy is as follows. Suppose there is a joint probability distribution:

(7) $$\begin{array}{c} P\left(X=x_{i},Y=y_{i}\right)=p_{ij},i=1,2,\ldots n;j=1,2,\ldots m \end{array}$$

Conditional entropy represents the uncertainty of the random variable Y given a known random variable X. Define the expectation of the entropy of the conditional probability distribution of Y for X when X is known: Conditional entropy quantifies the level of uncertainty associated with a random variable Y when the random variable X is specified. It is defined as the expected value of the entropy for Y’s conditional probability distribution, given the known values of X:

(8) $$\begin{array}{c} H\left(Y|X\right)=\sum_{i}^{n}p_{i}*H\left(Y|x_{i}\right)\\ \;p_{i}=P\left(X=x_{i}\right),i=1,\ldots n \end{array}$$

Information gain, denoted as g(D,A), for a feature *A* within a training set *D*, is characterized by the discrepancy between the empirical entropy of *D* and the empirical conditional entropy when *A* is present.

(9) $$\begin{array}{c} g(D,A)=H(D)-H(D|A) \end{array}$$

Here, H(D) is the empirical entropy of the dataset *D*, where *k* represents the number of categories of *Y*:

(10) $$\begin{array}{c} H\left(D\right)=-\sum_{k=1}^{k}\frac{\left|C_{k}\right|}{\left|D\right|}*\log_{2}\frac{\left|C_{k}\right|}{D} \end{array}$$

where $\left|C_{k}\right|$ is the number of instances in class *k*. And H(D∣A) is the empirical conditional entropy of *D* given *A*:

(11) $$H\left(D|A\right)=\sum_{i=1}^{N}\frac{|D_{i}|}{\left|D\right|}\cdot H\left(D_{i}\right)$$

where

(12) $$H\left(D_{i}\right)=-\sum_{k=1}^{K}\frac{|D_{ik}|}{|D_{i}|}\cdot \log_{2}\frac{|D_{ik}|}{|D_{i}|}$$

Here, $|D_{i}|$ is the number of instances in which feature *A* takes on the value corresponding to the *i*th subset, and $|D_{ik}|$ is the number of instances in subset *i* that belong to class *k*.

#### 3.3.3. Lightweight Model Construction

After dimensionality reduction, a new dataset is formed according to the optimal feature subset obtained in the previous step. According to the latest dataset, the model structure is adjusted and retrained to get the lightweight model. The validation set is used to evaluate the lightweight model. Since the model has gone through an explicable analysis, its robustness has been dramatically improved and is more in line with the needs of the actual scenario.

## 4. Experimental Setup

The experiment aimed to extract pertinent features from the initial dataset to create a more streamlined one. Python served as the coding language, and training was conducted using the RF and CNN algorithms provided by the Sklearn and Keras frameworks.

### 4.1. Datasets Description

The CICIDS2017 dataset, accessible to the public, serves as a repository for intrusion detection and prevention [35]. Captured within a specific subnet, it mirrors actual network traffic by undergoing exposure to prevalent, contemporary network attack techniques. It encompasses both benign and typical attack scenarios that echo those found in authentic PCAPs (packet capture files). Initially comprising more than 80 features, the dataset was refined by removing non-numeric attributes, preserving 78 features as the foundational dataset. Included in the attacks are malicious FTP, SSH, DOS, HeartBleed, web attack penetrations, botnet activities, and DDoS attacks. For balanced traffic classification data, eight varieties of malicious attacks were chosen in conjunction with standard traffic. The dataset specifically boasts 552,373 instances of attack data and 556,556 instances of normal traffic. Detailed information regarding particular attack types is available in Table 1. Moreover, the NSL KDD dataset [36] is made up of four distinct files: KDDTrain+.csv, KDDTrain±20Percent.csv, KDDTest+.csv, and KDDTest-21.csv. The NSL-KDD training set is designed to exclude repetitive records, thereby averting any bias in classifiers towards frequent instances, and the test set is structured to facilitate precise detection by removing duplicates.

The datasets kddtrain+.csv and kddtest+.csv were consolidated, with subsequent reprocessing and labeling of data into attack and normal types. The count of attack data stood at 71,445, while normal data comprised 77,054 entries. Initially, the dataset contained 45 features; however, after discarding a number of non-numeric features, 38 features were retained to serve as the foundational dataset for the training process.

### 4.2. Experimental Setting

Our experimental setting comprised an AMD2700X processor, 16 GB of RAM, an NVIDIA RTX2070 graphics card, and software environments including CUDA 7.5 and CUDNN 10.5. We used Python 3 as our programming language of choice. Detailed parameters of our setup can be found in Table 2. To classify malicious traffic, we performed comparative analyses using two different algorithms: CNN and RF. We allocated 50% to training for our dataset and the remaining 50% to testing.

### 4.3. Interpretation of NIDS Models

**Figure 5:**
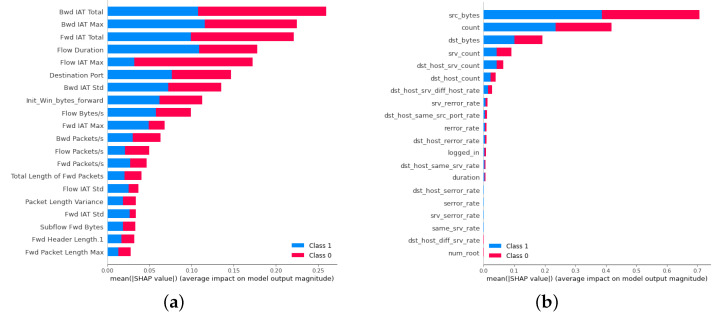
Interpretation results of CNN model. (**a**) Interpretation results of CNN model on CICIDS2017. (**b**) Interpretation results of CNN model on NSL-KDD.

**Figure 6:**
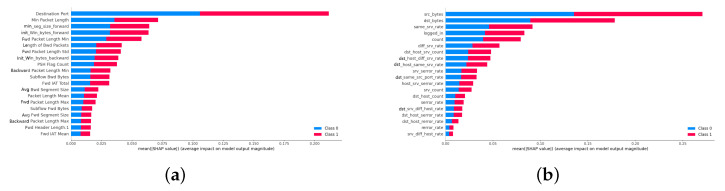
Interpretation results of RF model. (**a**) Interpretation results of RF model on CICIDS2017. (**b**) Interpretation results of RF model on NSL-KDD.

To confirm the interpretability of both models, we used the SHAP method for explanatory analysis. The contributions of the features identified by the models were ranked according to their impact on the application’s performance, confirming the models’ reliability. The interpretation results of CNN on CICIDS2017 is shown in Figure 5a, accordingly, CNN on NSL-KDD, RF on CICIDS2017 and RF on NSL-KDD are shown in Figure 5b, Figure 6a,b, respectively.

### 4.4. Feature Selection

Utilizing the identified key features, we proceeded with further training using CNN and RF. Regarding the CICIDS2017 dataset, from the initial 78 features, we tested subsets of 20, 15, and 10 features. Similarly, for the NSL-KDD dataset, we evaluated subsets of 20, 15, and 10 features from the original 38 features.

**Figure 7:**
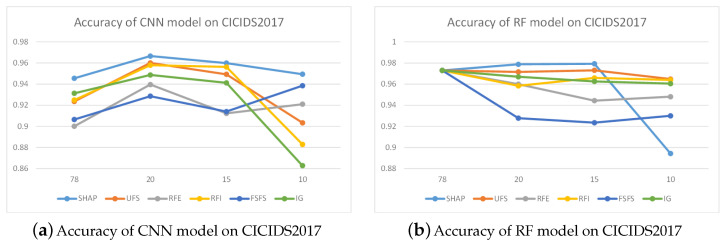
Detection accuracy on CICIDS2017.

**Figure 8:**
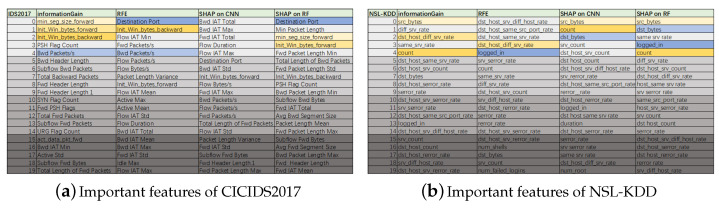
Important features of the datasets.

Figure 7 illustrates the performance accuracy of the CNN model when trained using various feature selection techniques on the CICIDS2017 dataset, in which all colors of curves indicate different feature selection methods. The figure reveals that with 20 selected features, the performance accuracy of the CNN models, as trained by the three distinct methods, was nearly identical, hovering around 98%. Reducing features to 15 led to a noticeable decrease in performance for the CNN (RFE) method, and further diminishing the feature count to 10 resulted in the CNN (RFE) method’s accuracy dropping to 92%, while the other two methods maintained an accuracy close to 98%. By integrating the outcomes from Ig and RFE, we prioritized the feature significance across both datasets, selecting the top 20 for inclusion in Figure 8.

The top 20 features derived from the NSL-KDD dataset revealed a substantial overlap with the critical features outlined in paper [37]. Moreover, among the top five features, 1–2 align with those identified by conventional feature selection techniques, validating the practicality of our proposed feature selection approach. Based on the feature contribution rankings, we reorganized the dataset and conducted recognition performance tests using CNN and RF, with detailed outcomes presented in Section 5.

## 5. Evaluation and Discussion

### 5.1. Detection Accuracy of NIDS Models

Leveraging the prominent features identified, we advanced the training with CNN and RF algorithms. The CICIDS2017 dataset initially comprised 78 features; we curated subsets of 20, 15, and 10 features for our experiments. Similarly, from the original 38 features of the NSL-KDD dataset, we extracted subsets of 20, 15, and 10 features to evaluate their efficacy.

Figure 7a depicts the precision of the CNN model when trained with varying feature selection methodologies on the CICIDS2017 dataset. The observation results indicated that the accuracy of CNN models trained using six feature selection methods improved when the number of features was reduced to 20. Reducing the number of features to 15 led to a decreasing trend in the accuracy of CNN methods, but it still remained higher than when there were 78 features. As the number of features was further reduced to 10, UFS, RFI, and IG showed a rapid decline in accuracy, while FSFS and RFE exhibited an upward trend in accuracy. Meanwhile, although SHAP’s accuracy decreased slightly, it still remained the highest among the six methods.

Figure 7b presents the accuracy of RF models trained with different feature selection methods on the CICIDS2017 dataset. SHAP, RFI and IG methods demonstrated relatively stable accuracy when reducing the number of features, while other methods such as UFS, RFE, and FSFS showed more significant performance degradation as the number of features decreased. When the number of features was reduced to 10, there was a slight decrease in model accuracy, which may be due to the fact that most other feature selection methods were based on random forest feature selection and had relatively good adaptability to random forest models.

**Figure 9:**
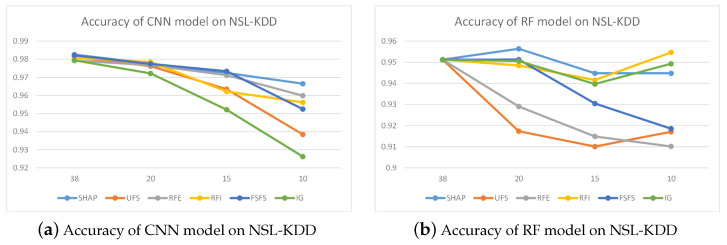
Detection accuracy on NSL-KDD.

Figure 9a presents the CNN model’s accuracy on the NSL-KDD dataset. The diagram indicates that even after applying the SHAP method to reduce features from 38 to 10, the model’s accuracy remained high at over 0.97. When other methods were used for feature selection, their accuracy was slightly lower than that of SHAP. Specifically, with feature counts of 20, 15, and 10, there was a noticeable drop in accuracy for other models, falling below 0.96, respectively. Overall, the SHAP method demonstrated superior performance.

The accuracy of the RF model on the NSL-KDD dataset is illustrated in Figure 9b. The UFS-based model exhibited the lowest accuracy with 15 and 20 features, while the SHAP method yielded nearly identical results. Meanwhile, at a feature count of 10, the RFI, IG, and SHAP methods’ models showed a slight increase in accuracy. Thus, the outcomes of the SHAP method were comparatively more favorable.

### 5.2. Resource Consumption of the NIDS Models

The subsequent figures draw upon two datasets, employing varying feature subsets and parameters derived from the CNN model training.

**Figure 10:**
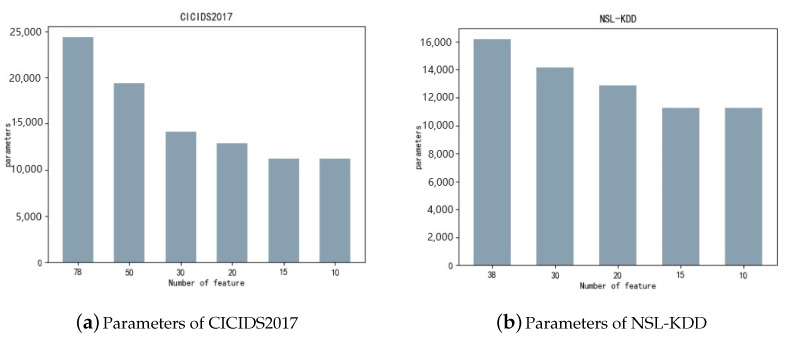
Number of model parameters.

Figure 10 demonstrates that reducing the parameter count to 10 can slash the model’s parameters by half to two-thirds. Furthermore, we conducted a comparative analysis of the datasets before and after selecting 10 features across multiple dimensions, such as training duration, inference latency, CPU usage, and post-training model dimensions. Each dataset underwent quintuple iterations to validate the experimental accuracy, yielding the ensuing data results.

**Figure 11:**
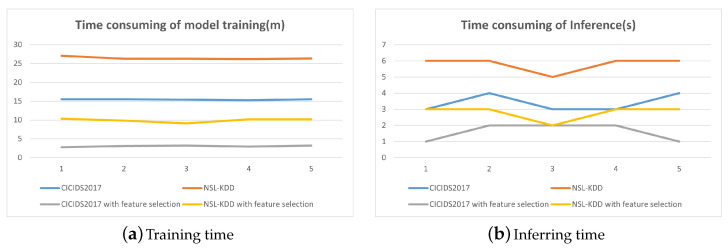
Training and inferring times.

Figure 11 demonstrates that the original dataset’s training times were approximately 15 and 27 min, with inference times around 6 and 3 s, respectively. Post-feature selection, these training times were reduced to about 3 and 10 min, and inference times to roughly 2 and 3 s, indicating a substantial decrease in time consumption.

From Figure 11a, we observe a marked reduction in model size. With the CICIDS2017 dataset, reducing features from 78 to 10 resulted in a model size that was one-third of the original. For the NSL-KDD dataset, cutting down features from 38 to 10 led to a model size of about half the initial size. This confirmed the efficacy of feature selection in diminishing model size. As depicted in Figure 11b, CPU usage for both datasets decreased from roughly 72% to about 50%. This reduction in feature count significantly lowered storage requirements and cut down on training and inference times, thereby enhancing model performance. Hence, selecting an optimal feature subset is crucial for the model’s practical deployment.

In the third phase, we present two figures illustrating the outcomes from two datasets, each employing distinct feature subsets and parameters derived from the CNN model training.

Figure 10 indicates that a reduction in parameters to 10 can decrease the model’s parameters by 50% to 66%. Moreover, we comprehensively compared the datasets, pre- and post-selection of 10 features, across several metrics such as training duration, inference time, CPU usage, and post-training model dimensions. To confirm the precision of our experiments, we conducted five trials for each dataset.

Figure 11 reveals that the training durations for the three datasets initially spanned approximately 31, 15, and 27 min, with inference times of roughly 7, 6, and 3 s, respectively. Following the application of feature selection, these training times were condensed to about 10, 3, and 10 min, and inference times to nearly 3, 2, and 3 s, respectively, indicating a notable reduction in temporal expenditure.

**Figure 12:**
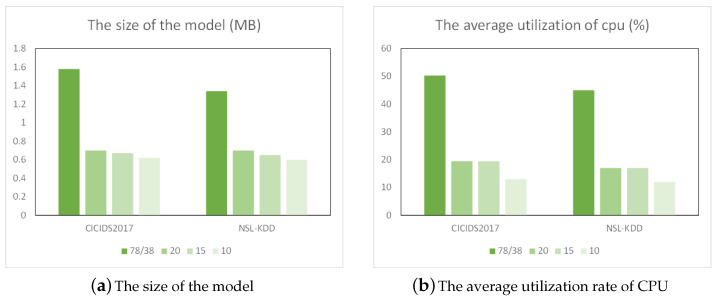
Resource consumption.

Observations from Figure 12a highlight a substantial alteration in model dimensions. With the CICIDS2017 dataset, the feature count was trimmed from 78 to 10, shrinking the model to a third of its original size. For the NSL-KDD dataset, the feature reduction from 38 and 39 to 10, respectively, halved the model size. This underscores the effectiveness of feature selection in minimizing model scale. As depicted in Figure 12b, CPU usage for all datasets decreased significantly from around 72% to approximately 50%.

**Figure 13:**
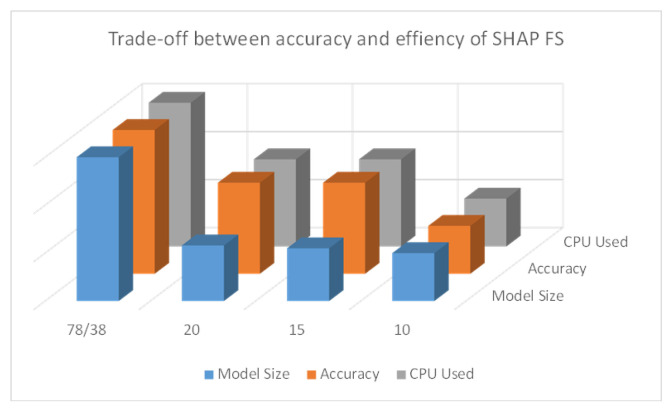
Trade-off between accuracy and efficiency of SHAP FS.

Figure 13 illustrates the trends in accuracy, CPU usage, and model size for the SHAP feature selection method proposed in this paper under varying numbers of features. There are three sets of data represented: accuracy, CPU usage, and model size. To facilitate the observation of the trends, the original values have been appropriately scaled to avoid issues with magnitude. From left to right on the x-axis: As the number of features decreases, information loss leads to a gradual decline in accuracy. The CPU usage and model size both show a decreasing trend. This indicates that in smaller models, while accuracy is affected, the computational resources required are also reduced, achieving a more lightweight model.

These findings underscore the importance of meticulous feature selection in enhancing our CNN model’s efficiency by curtailing storage requirements, training and inference times, and bolstering model performance. Thus, it underscores its critical role in the model’s practical deployment.

## 6. Conclusions and Future Work

Network intrusion detection systems are vital elements within network management and security, with deep learning standing out as a superior method for extracting high-dimensional features and playing an essential role in NIDSs. Despite this, the rapid evolution of network technologies has led to a surge in network traffic volumes. Deep learning models, known for their intricate structures and extensive parameters, face challenges such as packet loss and delays during numerous traffic detection tasks, which impede their ability to fulfill real-world demands. Feature selection is a potent strategy for reducing data dimensions and simplifying tasks, thereby boosting model performance. This study introduced an interpretable technique for feature selection in intrusion detection, explicitly tailored for analyzing encrypted traffic, aligning with the principles of causality.

Initially, we interpreted the original model to ascertain each feature’s influence. Following this, we sorted features by their level of impact and selected a subset that significantly aided the process, meeting the set criteria for an optimal subset. Subsequently, we refined an intrusion detection model with this chosen feature set for network traffic analysis. Our experiments, which included both innovative and established feature selection methods, utilized two models—a convolutional neural network (CNN) and a random forest (RF)—and two datasets (KDD-NSL and CICIDS2017) for validation. The findings confirmed that our method’s optimal feature subset sustained high accuracy in malicious traffic classification while streamlining the model. Notably, our approach secured over 98% accuracy, even with ten features in the screening. We reduced the dataset’s features from 78 to 10, halving the training/inference duration, and cutting model parameters by over 30%, significantly economizing storage. Feature selection thus markedly improved the classification efficacy of malicious traffic models.

Nonetheless, our current experiments are confined to offline feature extraction. Future endeavors will focus more on real-time network feature extraction and traffic categorization to ensure our findings are effectively transferable to practical scenarios.

## Figures and Tables

**Table 1:** Datasets’ description (NSL-KDD and CICIDS2017).

Label	Attack Type	Count
1	Normal flow	556,556
0	DDoS	128,025
0	DoS GoldenEye	10,293
0	DoS Hulk	230,124
0	DoS Slowhttptest	5499
0	DoS slowloris	5796
0	FTP-Patator	7935
0	PortScan	158,804
0	SSH-Patator	5897
**Number**	**Attack Type**	**Count**
1	Normal	77,054
0	DoS	53,563
0	Probe	14,088
0	R2L	3542
0	U2R	252

**Table 2:** Experimental environment parameters.

Category	Parameters
GPU	NVIDIA RTX2060S
Operating system	Win 10
CPU	AMD 2700X
CUDA version	7.5
CuDNN version	10.5

## Data Availability

Data are contained within the article.
